# Identification of Strain-Specific B-cell Epitopes in *Trypanosoma cruzi* Using Genome-Scale Epitope Prediction and High-Throughput Immunoscreening with Peptide Arrays

**DOI:** 10.1371/journal.pntd.0002524

**Published:** 2013-10-31

**Authors:** Tiago Antônio de Oliveira Mendes, João Luís Reis Cunha, Rodrigo de Almeida Lourdes, Gabriela Flávia Rodrigues Luiz, Lucas Dhom Lemos, Ana Rita Rocha dos Santos, Antônia Cláudia Jácome da Câmara, Lúcia Maria da Cunha Galvão, Caryn Bern, Robert H. Gilman, Ricardo Toshio Fujiwara, Ricardo Tostes Gazzinelli, Daniella Castanheira Bartholomeu

**Affiliations:** 1 Departamento de Parasitologia, Universidade Federal de Minas Gerais, Belo Horizonte, Brasil; 2 Centro de Ciências da Saúde, Universidade Federal do Rio Grande do Norte, Natal, Brasil; 3 University of California, San Francisco, San Francisco, California, United States of America; 4 Universidad Cayetano Heredia, Lima, Peru; 5 Johns Hopkins University, Baltimore, Maryland, United States of America; 6 Departamento de Bioquímica, Universidade Federal de Minas Gerais, Belo Horizonte, Brasil; 7 Centro de Pesquisas Rene Rachou – Fundação Oswaldo Cruz, Belo Horizonte, Brasil; 8 Division of Infectious Diseases and Immunology, University of Massachusetts Medical School, Worcester, Massachusetts, United States of America; University of Pittsburgh, United States of America

## Abstract

**Background:**

The factors influencing variation in the clinical forms of Chagas disease have not been elucidated; however, it is likely that the genetics of both the host and the parasite are involved. Several studies have attempted to correlate the *T. cruzi* strains involved in infection with the clinical forms of the disease by using hemoculture and/or PCR-based genotyping of parasites from infected human tissues. However, both techniques have limitations that hamper the analysis of large numbers of samples. The goal of this work was to identify conserved and polymorphic linear B-cell epitopes of *T. cruzi* that could be used for serodiagnosis and serotyping of Chagas disease using ELISA.

**Methodology:**

By performing B-cell epitope prediction on proteins derived from pair of alleles of the hybrid CL Brener genome, we have identified conserved and polymorphic epitopes in the two CL Brener haplotypes. The rationale underlying this strategy is that, because CL Brener is a recent hybrid between the TcII and TcIII DTUs (discrete typing units), it is likely that polymorphic epitopes in pairs of alleles could also be polymorphic in the parental genotypes. We excluded sequences that are also present in the *Leishmania major*, *L. infantum*, *L. braziliensis* and *T. brucei* genomes to minimize the chance of cross-reactivity. A peptide array containing 150 peptides was covalently linked to a cellulose membrane, and the reactivity of the peptides was tested using sera from C57BL/6 mice chronically infected with the Colombiana (TcI) and CL Brener (TcVI) clones and Y (TcII) strain.

**Findings and Conclusions:**

A total of 36 peptides were considered reactive, and the cross-reactivity among the strains is in agreement with the evolutionary origin of the different *T. cruzi* DTUs. Four peptides were tested against a panel of chagasic patients using ELISA. A conserved peptide showed 95.8% sensitivity, 88.5% specificity, and 92.7% accuracy for the identification of *T. cruzi* in patients infected with different strains of the parasite. Therefore, this peptide, in association with other *T. cruzi* antigens, may improve Chagas disease serodiagnosis. Together, three polymorphic epitopes were able to discriminate between the three parasite strains used in this study and are thus potential targets for Chagas disease serotyping.

## Author Summary

Serological tests are preferentially used for the diagnosis of Chagas disease during the chronic phase because of the low parasitemia and high anti-*T. cruzi* antibody titers. However, contradictory or inconclusive results, mainly related to the characteristics of the antigens used, are often observed. Additionally, the factors influencing variation in the clinical forms of Chagas disease have not been elucidated, although it is likely that host and parasite genetics are involved. Several studies attempting to correlate the parasite strain with the clinical forms have used hemoculture and/or PCR-based genotyping. However, both techniques have limitations. Hemoculture requires the isolation of parasites from patient blood and the growth of these parasites in animals or *in vitro* culture, thereby possibly selecting certain subpopulations. Moreover, the level of parasitemia in the chronic phase is very low, hindering the detection of parasites. Additionally, direct genotyping of parasites from infected tissues is an invasive procedure that requires medical care and hinders studies with a large number of samples. The goal of this work was to identify conserved and polymorphic linear B-cell epitopes of *T. cruzi* on a genome-wide scale for use in the serodiagnosis and serotyping of Chagas disease using ELISA. Development of a serotyping method based on the detection of strain-specific antibodies may help to understand the relationship between the infecting strain and disease evolution.

## Introduction

Chagas disease, a zoonosis caused by the protozoan parasite *Trypanosoma cruzi*, affects approximately 10 million people in the Americas. Approximately 14,000 deaths occur annually, and 50,000–200,000 new cases are diagnosed each year [1]. During the acute phase of infection, diagnosis is based on parasitological methods [2]; however, in the chronic phase, such parasitological approaches have a low sensitivity, between 50–65%, because of low levels of parasitemia [3], [4]. The chronic phase is also characterized by a strong and persistent humoral immune response, thus the measurement of IgG antibodies specific for parasite antigens should be performed for diagnosis [5]. However, serological methods from different laboratories have been observed to be inconclusive or contradictory [6]–[8]. These discrepancies are mainly related to technical errors and antigen composition because crude or semi-purified protein extracts of epimastigotes, a parasite stage not found in the mammalian host, are generally used [6], [9]. Moreover, false-positive results are frequently observed because of the cross-reactivity of crude preparations of *T. cruzi* antigens with sera from individuals infected with *Leishmania sp*. and *T. rangeli* [10]–[12]. The use of recombinant antigens and synthetic peptides as a substitute for parasite lysates has increased reproducibility and, in addition, does not require the maintenance and processing of live parasites [13], [14]. Despite recent advances in Chagas disease diagnostics, the methods available still have limitations related to low specificity and sensitivity [15], [16]. Among the factors that compromise the performance of diagnostic tests, the genetic variability of the parasite is known to contribute to false-negative results in Chagas disease serodiagnosis [17].

Epidemiological, biochemical, and molecular studies have demonstrated that the *T. cruzi* taxon is extremely polymorphic [18]–[21]. Recently, *T. cruzi* strains were reclassified into six DTUs (discrete typing units) called TcI to TcVI [22], and there is much speculation regarding whether this parasite variability could be associated with different disease prognoses. Although *T. cruzi* infection results in a broad spectrum of clinical forms as indeterminate, cardiac, and digestive forms, the determinant factors involved in the development of each clinical form have not been elucidated, though it is likely that genetic factors of the host and parasite are involved [23]. However, no study to date has found an unequivocal association between the infecting parasite DTU and the clinical forms of the disease. Nevertheless, this hypothesis has not been discarded because correlations between the geographic distribution profiles of different *T. cruzi* DTUs and a higher frequency of specific clinical forms have been reported [21]. Indeed, digestive manifestations are more common in the central region of Brazil and the southern part of South America, where infection by TcII, TcV, and TcVI predominates; in contrast, such manifestations are rare in the northern part of South America and in Central America, where infection caused by TcI is more common [24].

Correlation studies between the parasite DTU and clinical forms of Chagas disease are challenging because most of the techniques require parasite isolation from patient blood or parasite genotyping directly from infected tissues. Because many *T. cruzi* populations are polyclonal, hemoculture may select sub-populations of parasites more adapted to *in vitro* growth conditions [25]. Moreover, because of different tissue tropisms of some *T. cruzi* strains [26], in infections caused by polyclonal populations and/or co-infections, the clones circulating in the patient blood may not be the same as those found in tissue lesions. The current methodologies to genotype the parasite from tissue biopsies are laborious and expensive, thus limiting the number of samples that can be analyzed. Within this context, a parasite typing method based on the detection of strain-specific antibodies from patient sera could resolve many of these problems. Thus far, there is only one study that proposes the use of an antigen to discriminate among *T. cruzi* DTUs [17]. This study is based on an antigen named TSSA (trypomastigote small surface antigen), belonging to the TcMUC III protein family, which can differentiate between humans infected with TcI, TcIII, and TcIV and those infected with TcII, TcV, and TcVI.

In the present study, we performed a genomic screen to identify polymorphic and conserved linear B-cell epitopes in the predicted proteome of the CL Brener *T. cruzi* strain in an attempt to identify targets for the serotyping and serodiagnosis, respectively, of *T. cruzi*-infected patients. The results were validated using sera from experimentally infected mice and chagasic patients. 

## Materials and Methods

### Ethics statement

The design and methodology of all experiments involving mice were in accordance with the guidelines of COBEA (Brazilian College of Animal Experimentation), strictly followed the Brazilian law for “Procedures for the Scientific Use of Animals” (11.794/2008), and were approved by the animal-care ethics committee of the Federal University of Minas Gerais (protocol number 143/2009).

The study protocol involving human samples from Bolivia was approved by the ethics committees of the study hospital, A.B. PRISMA, Johns Hopkins University and the U.S. Centers for Disease Control and Prevention. All subjects provided written informed consent before blood was collected. As for the Brazilian patients, written informed consent was obtained from the participants and was approved by the Ethics Committee of the Federal University of Minas Gerais (UFMG), under protocol number No. 312/06.

### Mouse sera

Each experimental group was composed of six 2–4-week-old C57BL/6 male mice. The mice were infected with 50 Colombiana or 500 Y trypomastigotes. For the CL Brener clone, we used three mouse groups infected with 50, 100, or 500 trypomastigotes. Infection was confirmed by the observation of trypomastigote forms in blood collected from the tail at seven days after intraperitoneal inoculation. One additional group was infected with 1×10^5^ *T. rangeli* trypomastigotes, and the infection was confirmed by PCR [27]. Six un-infected mice were used as the control group. The chronic phase of infection was confirmed after approximately 3 months by negative parasitemia and the presence of anti-parasite IgG (as tested against *T. cruzi* and *T. rangeli* crude antigens) by ELISA [28]. Mouse blood was then obtained by cardiac puncture; coagulation was performed at room temperature for 30 minutes, and the serum was obtained after centrifugation at 4000×g for 15 minutes.

### Human sera

Blood samples from chagasic patients from Bolivia were collected in a public hospital in Santa Cruz de la Sierra. DNA was extracted from patient blood samples and parasite genotyping was performed as previously described [29]. Infection by TcI parasite lineage was confirmed for six samples (Supplementary Figure S1). Samples from 10 chagasic patients previously characterized to be infected with TcII [30] and 56 samples from chagasic patients infected with untyped parasites collected from Rio Grande do Norte State, Brazil, were also used. Samples from 14 patients infected with *L. braziliensis* and 14 patients with visceral leishmaniasis both known to be un-infected with *T. cruzi* and the sera from 24 un-infected humans were used as specificity and negative controls, respectively.

### Parasites

Epimastigotes of the Colombiana and CL Brener clones, and Y strain of *T. cruzi* and *T. rangeli* SC-58 were maintained in a logarithmic growth phase at 28°C in liver infusion tryptose (LIT) medium supplemented with 10% fetal bovine serum, 100 µg/mL streptomycin, and 100 units/mL penicillin [31]. A total of 1×10^6^ *T. cruzi* epimastigotes/mL were incubated in triatomine artificial urine (TAU) medium for 2 hours at 28°C. L-proline (10 mM) was added to the medium, and the metacyclic forms were obtained after 72 hours at 28°C [32]. Trypomastigotes and amastigotes were obtained from rhesus-monkey epithelial LLC-MK2 cells infected with metacyclic forms cultured in RPMI medium supplemented with 2% fetal bovine serum at 37°C and 5% CO_2_ [31]. Differentiation of *T. rangeli* epimastigotes to trypomastigotes was induced with 10^6^ parasites/mL in DMEM medium (pH 8) for 6 days at 28°C [33].

### *In silico* prediction of linear B-cell epitopes

Linear B-cell epitopes were predicted for all the proteins of the CL Brener genome release 4.1 [34] using the Bepipred 1.0 program with a cutoff of 1.3 [35]. The BepiPred program assigns a score to each individual amino acid in a sequence, therefore only amino acids with prediction Bepipred score ≥1.3 were considered for the downstream analysis. Proteins encoded by the pair of Esmo and Non-Esmo alleles were aligned using the CLUSTALW program [36], and each pair of amino acids aligned received a polymorphism score according to the following scale: 0 for identical amino acids; 1 for different amino acids with similar physical-chemical properties; 2 for a mismatch involving amino acids with dissimilar physical-chemical properties; and 3 for a gap position. A perl script based on a sliding window approach that uses a fixed window size of 15 amino acids and an increment of one amino acid identified all 15-mer subsequences in which each individual amino acid has a bepipred score ≥1.3. Those peptides with a polymorphism score above 6 (sum of the individual amino acid polymorphism scores) and a mean BepiPred score ≥1.3 were classified as polymorphic epitopes; those peptides identical between the Esmo and Non-Esmo haplotypes and with a mean BepiPred prediction score ≥1.3 were classified as conserved epitopes. The selected peptides were compared with the predicted proteins from the genomes of *L. infantum*, *L. major*, *L. braziliensis*, and *T. brucei* (release 4.1) [37] using the BLASTp algorithm [38]. Peptides with at least 70% similarity along 70% of the length were discarded. After elimination of peptides with potential cross-reactivity with *Leishmania* and *T. brucei*, 50 Esmo-like peptides, 50 Non-esmo-like peptides and 50 peptides conserved with the highest mean Bepipred score were selected.

### Spot synthesis and immunoblotting

Peptides were synthesized on pre-activated cellulose membranes according to the SPOT synthesis technique [39]. Briefly, Fmoc-amino acids were activated with 0.05 mM HOBt and 0.1 mM DIC and automatically spotted onto pre-activated cellulose membranes using the MultiPep SPOT synthesizer (Intavis AG). The non-binding sites of the membrane were blocked with 10% acetic anhydride, and the Fmoc groups were removed with 25% 4-methyl piperidine. These processes were repeated until peptide chain formation was complete. After synthesis, side-chain deprotection was performed by adding a 25∶25∶1.5∶1 solution of trifluoroacetic acid, dichloromethane, triisopropylsilane, and water. The amino acid coupling and side-chain deprotection were monitored by staining the membrane with 2% bromophenol blue. The immunoblotting methodologies followed a previously described protocol [39]. First, the membrane containing peptides was blocked with 5% BSA and 4% sucrose in PBS overnight and incubated with infected and control mouse sera diluted 1∶5,000 in blocking solution for 1 hour. After washing three times with PBS-T (PBS; 0.1% Tween 20), the membrane was incubated with the secondary HRP-conjugated anti-mouse IgG antibody (Sigma-Aldrich) diluted 1∶10,000 in blocking solution for 1 hour. After a third wash, detection was performed using ECL Plus Western blotting (GE Healthcare), following the manufacturer's instructions, with the Gel Logic 1500 Imaging System (Kodak). The densitometry measurements and analysis of each peptide were performed using Image Master Platinum (GE), and the relative intensity ratio (RI) cutoff for positivity was determined at 2.0.

### Soluble peptide synthesis

The soluble peptides were synthesized in solid phase on a 30-µmol scale using N-9-fluorenylmethoxycarbonyl [40] with PSSM8 equipment (Shimadzu). Briefly, Fmoc-amino acids were activated with a 1∶2 solution of HOBt and DIC. The active amino acids were incorporated into Rink amide resin with a substitution degree of 0.61. Fmoc deprotection was then performed using 25% 4-methylpiperidine. These steps were repeated until the synthesis of each peptide was complete. The peptides were deprotected and released form the resin by treatment with a solution of 9.4% trifluoroacetic acid, 2.4% water, and 0.1% triisopropylsilane. The peptides were precipitated with cold diisopropyl ether and purified by high-performance liquid chromatography (HPLC) on a C18 reverse-phase column using a gradient program of 0 to 25% acetonitrile. The peptides were obtained with 90% purity, as confirmed by mass spectrometry using Autoflex Speed MALDI/TOF equipment.

### ELISA and affinity ELISA

Each well of flexible ELISA polyvinylchloride plates (BD Falcon) was coated with 2 µg of soluble peptide. After blocking with 5% BSA in PBS for 1 hour at 37°C, followed by three washing steps with PBS containing 0.05% Tween 20 (PBS-T), the plates were incubated with human or mouse serum (dilution 1∶100). The plates were washed three times with PBS-T, and secondary HRP-conjugated anti-human or anti-mouse IgG antibody was added for 1 hour at 37°C, followed by four washes. A solution containing 0.1 M citric acid, 0.2 M Na_2_PO_4_, 0.05% OPD, and 0.1% H_2_O_2_ at pH 5.0 was used for detection; the reaction was stopped with 4 N H_2_SO_4_, and the absorbance was measured at 492 nm. The mean optical density value at 492 nm plus three times the standard deviation of the negative serum was used as the cutoff value. For affinity ELISA, 6 M urea was added for 5 min at 37°C after incubation with the primary antibodies; the remainder of the protocol was the same [41]. The results are shown as an affinity index (AI) determined as the ratio between the absorbance values of the samples treated and not treated with urea. An AI value lower than 40% represented low-affinity antibodies, between 41 and 70% was classified as intermediate affinity and higher than 70% as high affinity.

### DNA extraction and sequencing

Genomic DNA extraction was performed using the GFX^TM^ Genomic Blood DNA Purification kit (GE Healthcare) following the manufacturer's instructions. The DNA samples were quantified using a NanoDrop Spectrophotometer ND-1000 (Thermo Scientific). The PCR products amplified with the primers listed in Supplementary Table S1 were subjected to sequencing at both ends using the ABI Prism 3730×l DNA Analyzer (Applied Biosystems) by Macrogen Inc (Korea).

### RNA extraction and cDNA synthesis

Total RNA was isolated from 10^8^ epimastigotes, 10^6^ trypomastigotes, and 10^8^ LLC-MK2 cells infected with approximately 10^5^ intracellular amastigotes of the Colombiana, Y, and CL Brener strains using the NucleoSpin RNA II RNA extraction kit (Macherey-Nagel) following the procedures described by the manufacturer. RNA from 10^8^ LLC-MK2 cells was also extracted and used as a negative control. The concentration and purity of the RNA samples were measured with a NanoDrop Spectrophotometer ND-1000 (Thermo Scientific). cDNA was synthesized using 10 ng of total RNA and the High Capacity cDNA Reverse Transcription Kit (Applied Biosystems, Foster City, CA) using random hexamer primers according to the manufacturer's instructions.

### Real-time PCR

Specific primers for each Esmo and Non-Esmo allele were designed, and the primer specificity was verified by electronic PCR using the entire parasite genome as a template. The primers used are listed in the Supplementary Table S2. Real-time PCR reactions were performed in an ABI 7500 sequence detection system (Applied Biosystems). The reactions were prepared in triplicate and contained 1 mM forward and reverse primers, SYBR Green Master Mix (Applied Biosystems), and 20 ng of cDNA. Standard curves were prepared for each experiment for each pair of primers using serially diluted *T. cruzi* CL Brener genomic DNA to calculate the relative quantity (Rq) values for each sample. qRT-PCRs for the constitutively expressed GAPDH gene were performed to normalize the expression of the specific alleles.

### Statistical analysis

All statistical analyses were performed using Graph Prism 5.0 software. First, the normal distribution of data was evaluated by the Kolmogorov-Smirnov test; because all they showed a Gaussian profile, an unpaired t test was used for the comparative analysis between the two sets of data, and an ANOVA was used for three or more experimental groups. P-values lower than 0.05 were considered statistically significant. The sensitivity, specificity, and accuracy of the peptides were also calculated for the human samples. The sensitivity is represented by Se = TP/(TP+FN), where TP (true positive) is the number of sera from individuals infected with *T. cruzi* above the cutoff value and FN (false negative) is the number of sera from infected individuals below the cutoff for the conserved peptide. For the polymorphic peptides, TP was defined as the number of sera from individuals infected with a specific strain above the cutoff value, and FN is the number of these sera below the cutoff for polymorphic peptides. The specificity is represented by Sp = TN/(TN+FP), where TN (true negative) is the number of sera from individuals infected with *L. braziliensis* or un-infected individuals below the cutoff and FP (false positive) is the number of sera from these samples with reactivity the for conserved peptide. For the polymorphic peptides, TN was defined as the number of sera from individuals infected with a non-specific strain or *L. braziliensis* and uninfected individuals below the cutoff, and FP is the number of sera from these samples with reactivity. The accuracy is calculated as Ac = (TP+TN)/(TP+TN+FP+FN).

## Results

### Epitope prediction using the *T. cruzi* CL Brener proteome and immunoblotting screening

We performed B-cell epitope prediction for 3,983 proteins derived from pair of alleles of CL Brener genome. We decided to restrict our analysis to this dataset because the CL Brener clone is a recent hybrid between the TcII and TcIII DTUs and evidence suggests that the latter is an ancient hybrid between TcI and TcII [42]. Therefore, it is likely that polymorphic epitopes in the pairs of alleles of CL Brener could also be polymorphic for its parental genotypes and other *T. cruzi* strains. In the CL Brener hybrid diploid genome, it is possible to identify two haplotypes: “Esmo”, which is more similar to TcII; and “Non-Esmo”, which is more similar to TcIII [34]. A total of 1,488 predicted epitopes were classified as conserved between the two haplotypes, and 428 were classified as polymorphic. We next excluded epitopes also present in *Leishmania major*, *L. infantum*, *L. braziliensis* and *T. brucei* to minimize the chance of cross-reactivity, because these parasites share many antigens with *T. cruzi* [10], [12], [16], thus reducing the number of conserved and polymorphic epitopes to 1,086 and 242, respectively.

**Figure 1:**
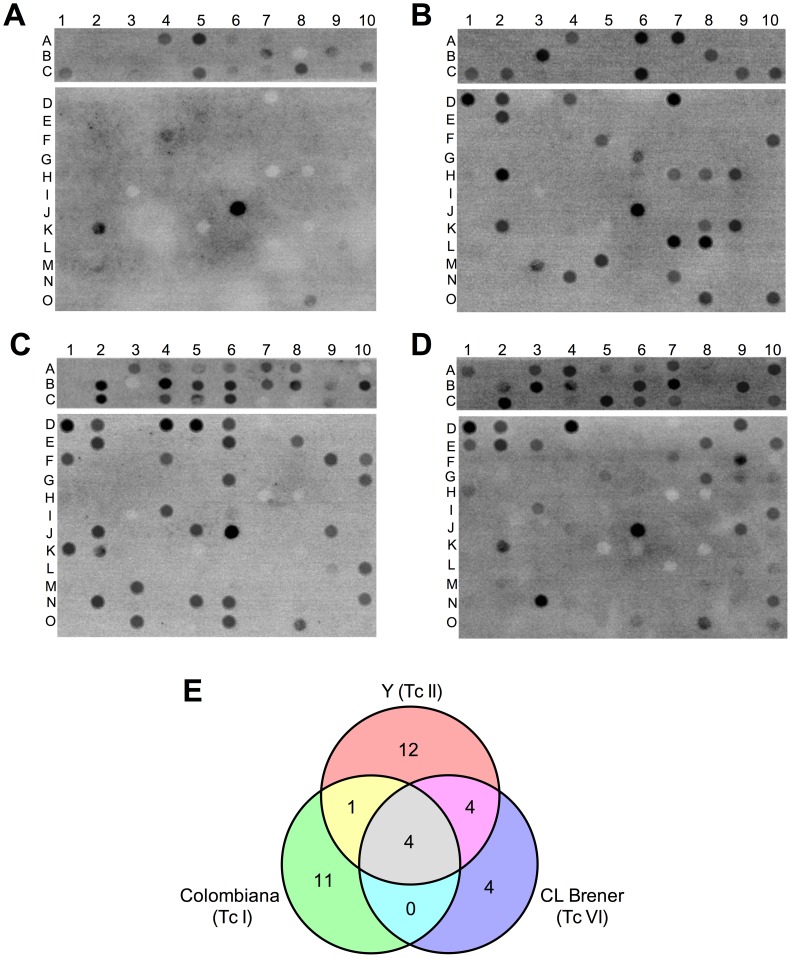
Immunoscreening of peptide arrays with sera from mice infected with different *T. cruzi* strains. (A) Sera from un-infected mice; sera from mice infected with Colombiana (TcI) (B), Y (TcII) (C), or CL Brener (TcVI) (D). (E) Venn diagram showing the specific and shared epitopes among *T. cruzi* strains.

A total of 50 conserved, 50 polymorphic Esmo-specific, and 50 polymorphic Non-Esmo-specific peptides with high epitope prediction scores were selected for the construction of peptide arrays. The reactivity of the peptides was tested using a pool of sera from six C57BL/6 mice chronically infected with Colombiana (TcI), Y (TcII), or CL Brener (TcVI) strains and un-infected mice as the control group (Figure 1). The quantification of the reactivity was performed by densitometric analysis (Supplementary Table S3). A peptide was considered reactive and antigenically conserved if its intensity signal with all *T. cruzi* strains was two times higher than its signal with the sera from un-infected mice. A peptide was considered reactive and antigenically polymorphic if its intensity signal with a specific strain was two times higher than the values with the other two strains and the un-infected mice. A total of 36 peptides were considered reactive with at least one strain (Figure 1E). A conserved peptide with the highest reactivity with all *T. cruzi* strains (C6_30_cons) and three polymorphic peptides specific for Colombiana (A6_30_col), Y (B2_30_y), and CL Brener (B9_30_cl) were selected for soluble synthesis, and their reactivity was validated by ELISA.

### Epitope validation with ELISA and affinity ELISA

**Figure 2:**
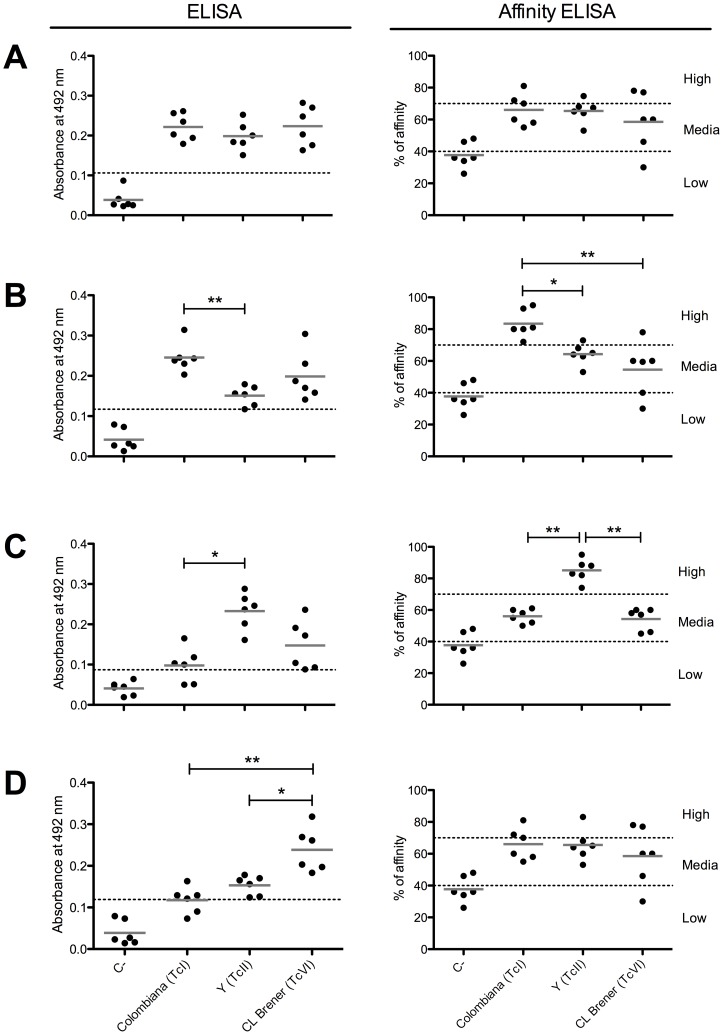
Reactivity and affinity of sera from *T. cruzi*-infected mice against conserved and polymorphic epitopes. (A) Peptide C6_30_cons. (B) Peptide A6_30_col. (C) Peptide B2_30_y. (D) Peptide B9_30_cl. The dotted line represents the cutoff value. The solid gray line represents the mean values. C-, un-infected mice. Colombiana (TcI), mice infected with the Colombiana strain. Y (TcII), mice infected with the Y strain. CL Brener (TcVI), mice infected with the CL Brener strain. *p<0.05 and **p<0.005.

Because immunoblotting assays are semi-quantitative techniques, we validated the results with quantitative ELISA and affinity ELISA assays using individual sera from six C57BL/6 mice chronically infected with the Colombiana (TcI), Y (TcII), or CL Brener (TcVI) strains and the un-infected mice as a control group. For the conserved peptide C6_30_cons, no significant difference in the reactivity among the sera from animals infected with different *T. cruzi* strains was observed (Figure 2A). The sera from mice infected with the Colombiana strain had a higher antibody titer against the A6_30_col peptide compared to the sera from mice infected with the Y strain. More importantly, the affinity antibodies discriminated Colombiana infection from those caused by the other two strains (Figure 2B). An expected recognition profile was also observed for the peptide B2_30_y (Figure 2C): sera from mice infected with the Y strain had a significantly higher antibody titer than those from mice infected with Colombiana, and the highest affinity antibodies generated by the Y strain discriminated its infection from those caused by Colombiana and CL Brener. With regard to peptide B9_30_cl, conventional ELISA was able to discriminate CL Brener infection from that caused by Y and Colombiana (Figure 2D).

Because the infection caused by different *T. cruzi* strains has specific evolution and mortality rates in a mouse model [43], we infected mice with a distinct parasite inoculum for each strain to reach the chronic phase when the sera were collected. Thus, to evaluate whether the differences in reactivity observed in the ELISA experiments were dependent on the inoculum, we tested the reactivity of sera from mice infected with 50, 100, or 500 CL Brener trypomastigotes (Supplementary Figure S2). There was no significant variation among the different CL Brener inocula, suggesting that the antigenic variability among the parasite strains is the main factor responsible for the distinct recognition profile of the peptides tested in the ELISA experiments. The evaluation of cross-reactivity with sera from mice infected with *T. rangeli* and from Leishmaniasis patients demonstrated that the peptides are *T. cruzi* specific (Supplementary Figures S3 and S4).

### Prediction of specific epitope reactivity with different *T. cruzi* DTUs

**Figure 3:**
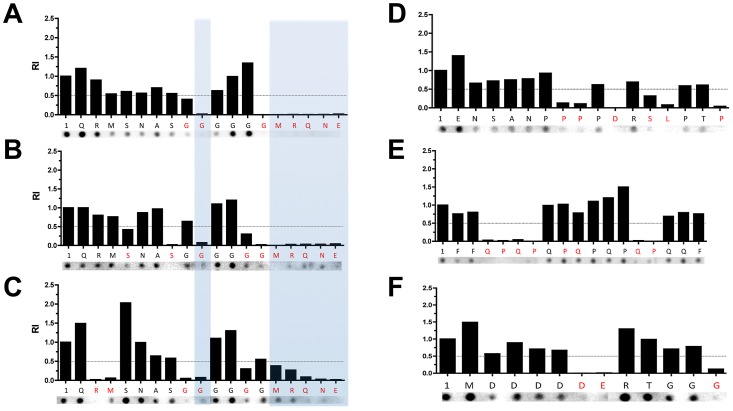
Mapping of antibody binding sites on the conserved and polymorphic epitopes by Ala-Scan. Reactivity of peptide C6_30_cons against the sera from mice infected with Colombiana (A), Y (B), and CL Brener (C). Reactivity of peptide A6_30_col against the sera from mice infected with the Colombiana clone (D). Reactivity of peptide B2_30_y against the sera from mice infected with the Y strain (E). Reactivity of peptide B9_30_cl against the sera from mice infected with the CL Brener clone (F). 1, original peptide. RI, relative intensity calculated by the ratio between the reactivity of the peptide with a specific amino acid substitution and the original peptide. The dotted line indicates half of the intensity value obtained with the original peptide. The red letters represent amino acid substitutions that reduce the reactivity to at least half of the value obtained with the original peptide. The blue squares show the conserved amino acids critical for antibody binding with the sera from mice infected with different *T. cruzi* strains.

We next analyzed the polymorphisms of the epitopes identified in this study and predicted their reactivity with sera from individuals infected with *T. cruzi* strains representative of each DTU (TcI to TcVI). To this end, we first subjected the peptide sequences to an AlaScan analysis [44] to identify the amino acid residues critical to antibody binding. We found that the pattern GXXXXMRQNE in the carboxy-terminal region of conserved peptide C6_30_cons is important for the interaction with the antibodies generated in infection caused by the three *T. cruzi* strains (Figures 3A, B, and C). As for the polymorphic epitopes, the patterns PPXDXSLXXP in peptide A6_30_col (Figure 3D), QPQPXPQXXXQP in B2_30_y (Figure 3E), and DEXXXXG in B9_30_cl (Figure 3F) are critical for binding with the antibodies generated by Colombiana, Y, and CL Brener infections, respectively.

**Figure 4:**
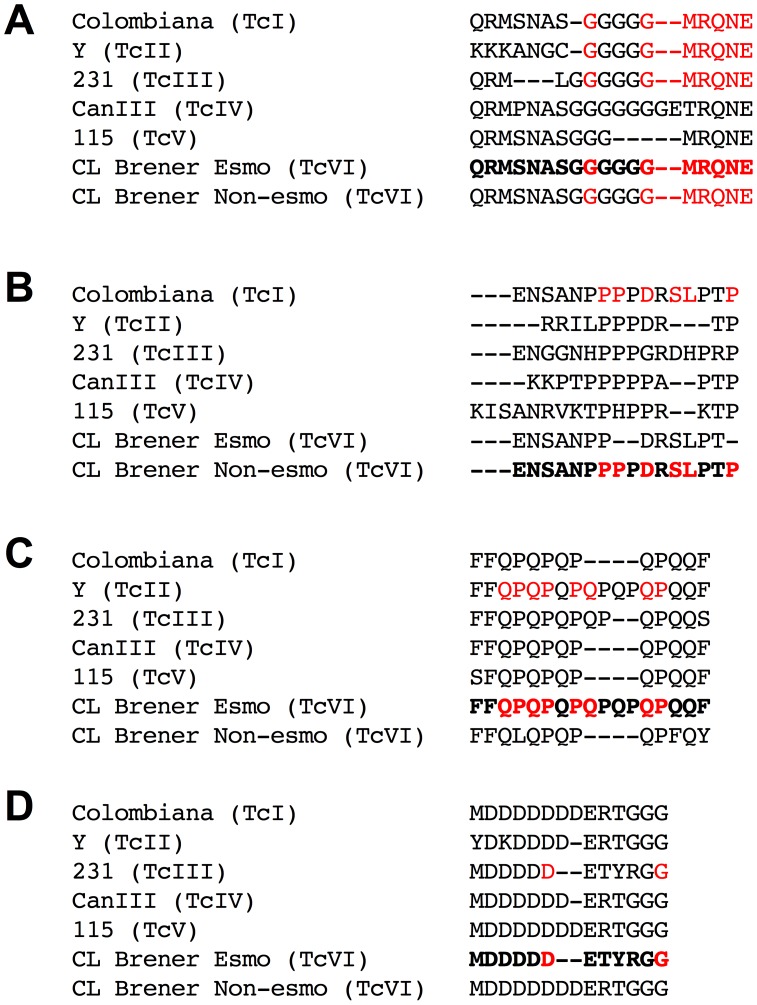
Reactivity prediction of epitopes based on the sequences encoding the identified peptides in different *T. cruzi* strains. (A) Peptide C6_30_cons. (B) Peptide A6_30_col. (C) Peptide B2_30_y. (D) Peptide B9_30_cl. The bold sequences represent the synthetic peptide tested. The red letters represent key amino acids for antibody binding.

We then sequenced the genomic DNA encoding these four epitopes in strains representative of the six *T. cruzi* DTUs to predict whether the peptides would be recognized in infections caused by different parasite DTUs. It is expected that a peptide would be recognized in infections caused by a specific strain if the amino acid residues critical for antibody recognition are encoded by its genome. Based on this criterion, we predicted that conserved peptide C6_30 would be able to identify infection caused by four of the six *T. cruzi* DTUs (Figure 4A), whereas peptides A6_30_col and B2_30_y are expected to identify infections caused only by TcI and TcVI (Figure 4B) and TcII and TcVI (Figure 4C), respectively. Interestingly, the A6_30_col and B2_30_y epitopes are identical to the Non-Esmo- and Esmo-like CL Brener haplotypes, respectively, reinforcing the hypothesis that the nature of the CL Brener hybrid may have contributions of both the TcI and TcII genomes. B9_30_cl is predicted to identify patients infected with TcIII or TcVI (Figure 4D).

### Epitope expression at different parasite stages and strains

**Figure 5:**
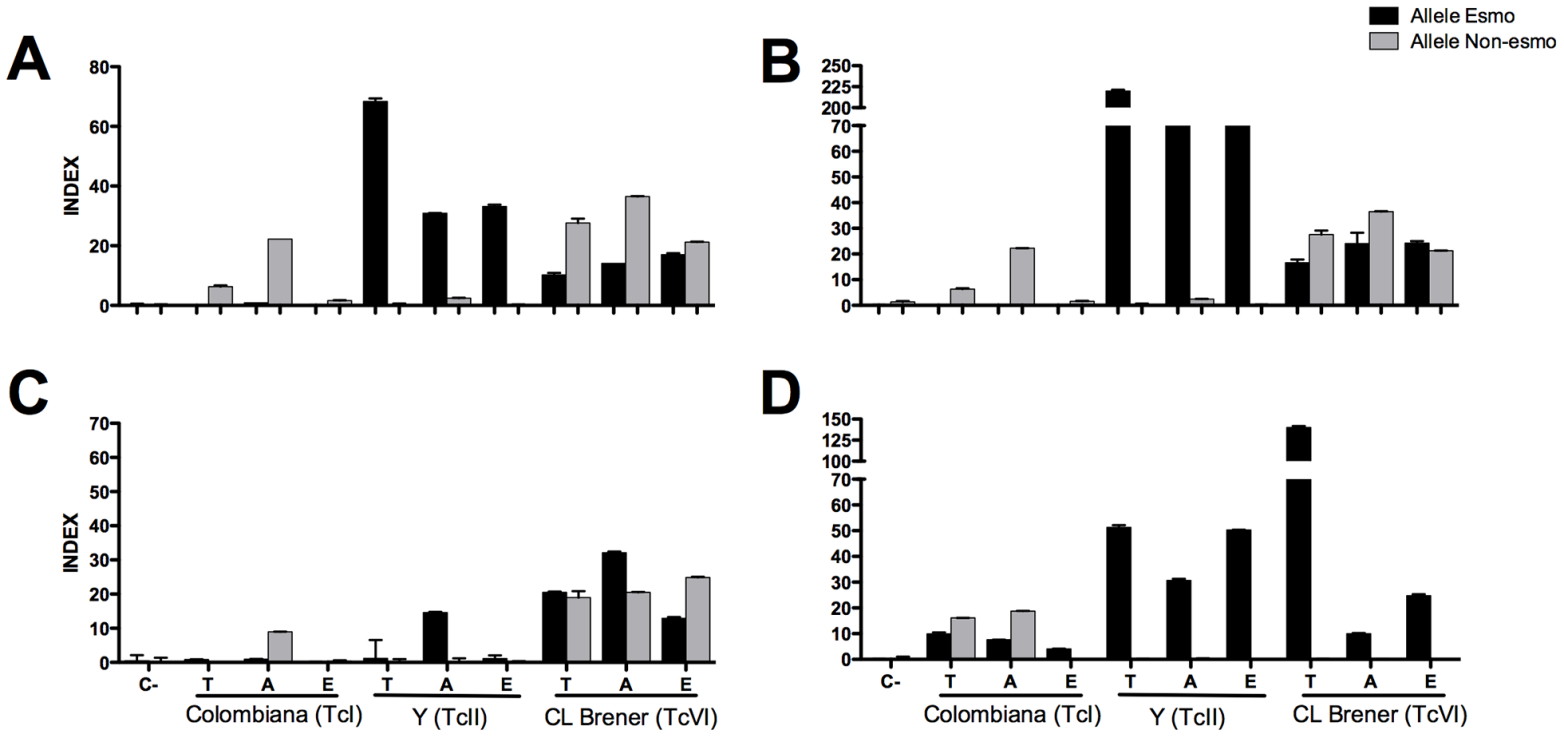
Expression levels of allele-specific genes encoding the identified conserved and polymorphic peptides. (A) Peptide C6_30_cons. (B) Peptide A6_30_col. (C) Peptide B2_30_y. (D) Peptide B9_30_cl. INDEX was calculated by the copy number from the mRNA level of each gene normalized by the GAPDH mRNA level. C-, negative control. T, trypomastigote. A, amastigote. E, epimastigote. The alleles were classified based on the annotation of the hybrid CL Brener genome.

Although all peptides are derived from the CL Brener genome, the sera from mice infected with this strain had lower antibody affinities for the A6_30_col and B2_30_y peptides than did the sera from mice infected with the Colombiana or Y strain (Figure 2). Because CL Brener is a hybrid strain [34], [42], [45], the polymorphic epitopes encoded by its pairs of alleles may have distinct expression levels that could explain the differences in their reactivity. To investigate this further, we designed allele-specific primers for the genes that encode the epitopes to evaluate their expression levels in the trypomastigote and amastigote forms, the parasite stages found in mammalian hosts (Figure 5). As expected based on the *T. cruzi* phylogeny [46], Y expressed only the Esmo-like variants, and Colombiana expressed only the Non-Esmo variants, except for the B9_30 transcript. CL Brener expressed both alleles of all genes, except for the B9_30 transcript. The conserved peptide was expressed by both the Esmo and Non-Esmo haplotypes of CL Brener (Figure 5A). The polymorphic Non-Esmo peptide A6_30_col was expressed by the Colombiana and CL Brener strains (Figure 5B), and CL Brener also expressed the Esmo-like allele for this peptide. The opposite profile was observed for the polymorphic Esmo B2_30_y peptide, whereby only the Y and CL Brener strains expressed the Esmo-like allele and CL Brener also expressed the Non-Esmo allele of this peptide (Figure 5C). CL Brener only expressed the Esmo-like variant of the B9_30_CL epitope, and its level of expression was approximately 5 times higher than in the Y strain (Figure 5D).

### Potential use of epitopes for human serodiagnosis and serotyping

**Figure 6:**
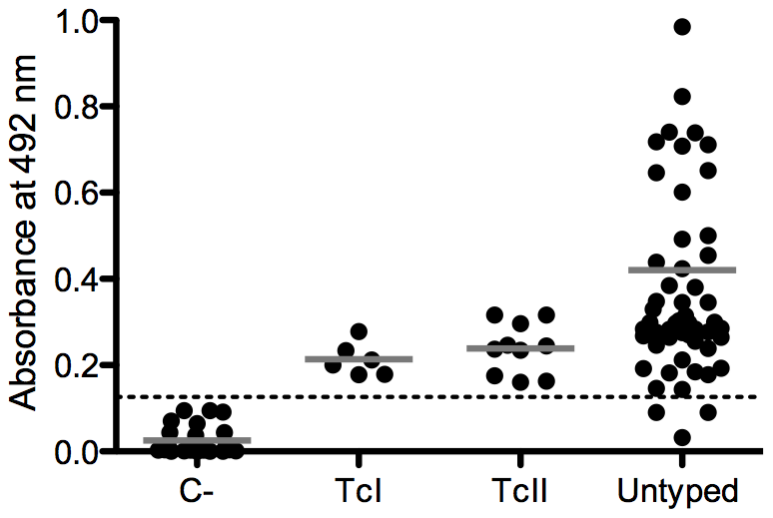
Reactivity of sera from humans infected with *T. cruzi* against the conserved peptide C6_30_cons. The dotted line represents the cutoff value. The solid gray line represents the mean values. C-, uninfected human. TcI, Chagasic patients infected with TcI DTU. TcII, Chagasic patients infected with the TcII DTU. Untyped, Chagasic patients infected with untyped parasites.

**Figure 7:**
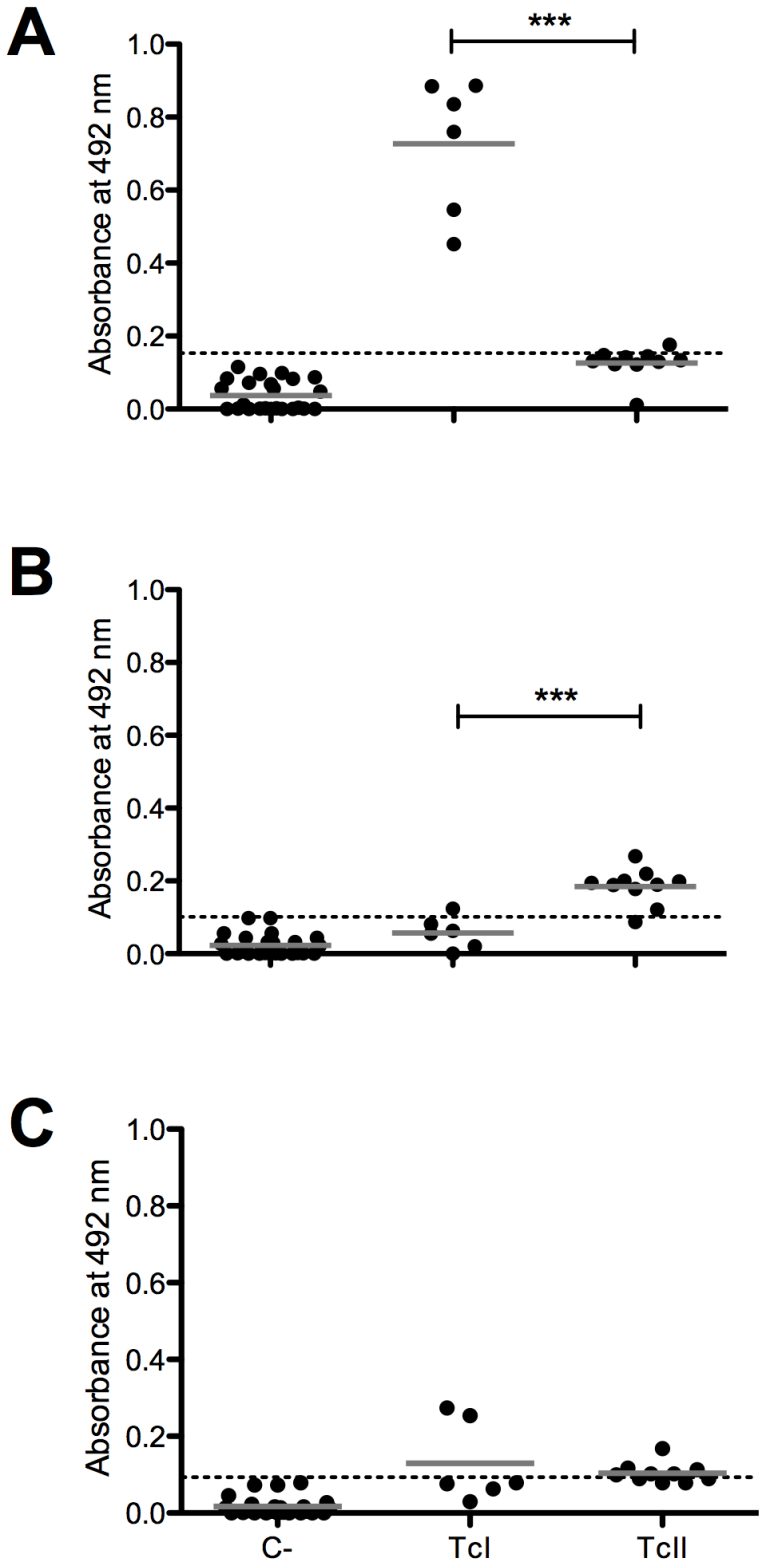
ELISA with sera from humans infected with TcI and TcII *T. cruzi* DTUs against the polymorphic peptides. (A) Peptide A6_30_col. (B) Peptide B2_30_y. (C) Peptide B9_30_cl. The dotted line represents the cutoff value. The solid gray line represents the mean values. C-, uninfected human. TcI, Chagasic patients infected with TcI DTU. TcII, Chagasic patients infected with the TcII DTU. ***p<0.001.

All previous results were based on a mouse model because the amount of the inoculum, infective strain, and time of infection can be adequately controlled. To test the potential application of these peptides for serodiagnosis and serotyping of human infection, we performed ELISA experiments with sera from chagasic patients with parasites genotyped as TcI or TcII, and healthy individuals. The conserved peptide C6_30_cons showed 95.8% sensitivity, 88.5% specificity, and 92.7% accuracy for the identification of chagasic patients, and no significant differences in the reactivity of sera from patients infected with TcI or TcII was observed (Figure 6). As expected, peptide A6_30_col showed much higher reactivity with the sera from patients infected with TcI (Figure 7A), with 100% sensitivity, 91.9% specificity, and 92.6% accuracy; peptide B2_30_y identified most of the individuals infected with TcII (Figure 7B), with 80% sensitivity, 94.8% specificity, and 92.6% accuracy. Additionally, none of the sera from patients infected with TcI recognized the B2_30_y peptide, and peptide B9_30_cl showed a low reactivity with both TcI and TcII (Figure 7C). All peptides were also *T. cruzi* specific because the majority of the sera from the patients infected with *L. braziliensis* were non-reactive (Supplementary Figure S4).

## Discussion

Despite efforts to identify new targets for the immunodiagnosis of Chagas disease, the impressive genetic variability of *T. cruzi* strains has imposed serious limitations on the development of high-sensitivity methods [47]–[50]. Additionally, serological cross-reactivity with *Leishmania* and *T. rangeli* infections [11], [12], [16] compromises the specificity of Chagas disease diagnosis. Therefore, the identification of new *T. cruzi*-specific antigens conserved among the parasite strains has been recognized as an important research area for Chagas disease diagnosis and control [47]. The polymorphic nature of *T. cruzi* isolates, on the other hand, opens new avenues for the development of serotyping methodologies to identify the parasite DTU causing infection based on a serological survey. For instance, this would allow large-scale epidemiological studies aimed at correlating the strain causing the infection with the clinical forms of Chagas disease, an open question that has been hampered by the limited number of samples that can be analyzed by the current genotyping methodologies [48]. To the best of our knowledge, only one study has identified a polymorphic epitope among the *T. cruzi* DTUs [49]. This marker is an immunodominant B-cell epitope of TSSA (trypomastigote small surface antigen), a representative of the TcMUC III gene family. The TSSA-I and TSSA-II isoforms serologically discriminate between animals infected with *T. cruzi* I from those infected with *T. cruzi* II, according to the previous DTU classification (TcII-VI in the current classification), respectively. In a serological survey of chagasic patients from Argentina, Brazil, and Chile, anti-TSSA antibodies recognized only the TSSA-II isoform, suggesting that the TcII-VI DTUs are the cause of Chagas disease in those regions. In a more recent study, however, this same research group analyzed the diversity of the TSSA gene in several representatives of each of the six *T. cruzi* DTUs and found a complex pattern of sequence polymorphism. Based on their analysis, the epitope considered to be specific for TcII-VI was shown to identify the TcII, V, and VI DTUs. In addition, the peptide previously described as TcI specific shares key features with TcIII and IV. Therefore, there is no *T. cruzi* DTU-specific serological marker identified thus far.

The goal of this work was to identify conserved and polymorphic linear B-cell epitopes of *T. cruzi* for Chagas disease serodiagnosis and serotyping using ELISA. This technique was selected because it is a quantitative assay and easily automated, thus allowing the analysis of a large number of samples. In recent years, synthetic peptides used as antigens have shown high sensitivity and specificity in diagnostic tests [50]. Peptides have several advantages over chemically purified or recombinant antigens because their production does not involve the manipulation of living organisms and can be obtained with a high level of purity [51]. Recently, the use of peptide arrays has allowed the immunoscreening of a large number of epitope candidates [39]. Thus, an approach based on a synthetic peptide array was chosen to screen of a large number of potential antigens by immunoblotting, followed by ELISA validation.

Initially, we screened the CL Brener genome to predict epitopes that are polymorphic and conserved between the Esmo and Non-Esmo haplotypes. The rationale underlying this strategy is that, because the CL Brener strain is a recent hybrid between the TcII and TcIII DTUs and there is evidence suggesting that the latter is an ancient hybrid between TcI and TcII [46], it is likely that the polymorphic epitopes between the CL Brener alleles would also be polymorphic among distinct *T. cruzi* strains. The Colombiana (TcI) and CL Brener (TcVI) clones and Y (TcII) strain were selected for this study to evaluate the degree of polymorphism of epitopes in TcII, a direct representative of one CL Brener parental DTU, and TcI, a more distant DTU of CL Brener, along with CL Brener.

The immunoscreening of 150 high-scoring peptides resulted in the identification of 36 novel epitopes, indicating that our computational approach for the prediction and prioritization of epitope candidates was successful. Our rate of success (24%) was slightly higher than previously described (19.5%) for *T. cruzi* using a similar validation approach [50]. We found that only 11% (4/36) of the reactive peptides are shared among the three parasite strains (Figure 1E), highlighting the problem with identifying high-sensitivity antigens for the serodiagnosis of Chagas disease due to the high degree of *T. cruzi* polymorphism. One of the conserved epitopes identified in this study, peptide C6_30_cons, has proven to be a new conserved *T. cruzi* antigen with a potential application in Chagas disease serodiagnosis (Figures 2A, 6A, S2, and S3).

Together, the three polymorphic epitopes were able to discriminate among infections caused by the three different *T. cruzi* strains included in this study and, thus, have the potential to be used for the serotyping of infections caused by this parasite. ELISA experiments using human sera confirmed the predictive reactivity of A6_30_col and B2_30_y (Figure 6). A6_30_col was able to identify 100% of the patients infected with TcI. As expected, the serum samples obtained from Brazilian patients known to be infected with TcII were reactive only with the C6_30 conserved and B2_30_y peptides. These results confirm the potential use of this peptide set for Chagas disease serotyping.

The peptide A6_30_col and B9_30_cl are derived from RNA binding proteins and RNA polymerase III, respectively (Supplementary Table S3). Both are predicted to have an intracellular localization. Indeed, humoral response against intracellular antigens is quite common in trypanosomatids as shown by the work described by da Rocha et al., 2002 [52] that performed immunoscreening of an amastigote cDNA library using sera from chagasic patients. About 70% of the amastigote antigens identified in this study is derived from intracellular parasite proteins. Similar to *Leishmania* infection, it is postulated that during *T. cruzi* infection a proportion of trypomastigotes/amastigotes cells are destroyed, thus releasing substantial amounts of multicomponent complexes containing intracellular antigens [53]. This reactivity could be the result of high abundance of these antigens as circulating complexes during the parasite infection due to high and constant expression of nuclear and house-keeping genes; higher stability due to formation of nucleoprotein particles more resistant to degradation; and their increased capacity to be processed by antigen-presenting cells because multicomponent particles are taken into the cell more efficiently than soluble antigens [54].

It is worth noting that the conserved and polymorphic epitopes identified in this study encompass repetitive regions. Interestingly, two of these peptides have proline-rich regions (Figure 4) that may be involved in protein-protein interactions in prokaryotes [55] and eukaryotes [56]. It has been demonstrated that the overall immunogenicity of proteins harboring tandem repeats is increased, as is the antigenicity of epitopes contained within repetitive units [52], [57]. Therefore, one expects that repeats receive a high B-cell epitope prediction score. Furthermore, the polymorphic epitopes containing repeats were top ranked for an additional reason: our polymorphic scale applied to the CL Brener pair of alleles attributes the highest score to a gap position in the alignment, a situation always present when the contraction or expansion of a repetitive region occurs in one sequence but not in another. This criterion was used because it is well known that repetitive sequences evolve faster than other regions of the genome [58], hence it is expect that they display a high level of polymorphism among distinct parasite strains. Additionally, because it is known that the number of repetitive antigenic motifs may affect antibody binding affinity [59], we hypothesized that polymorphic repetitive epitopes would be differentially recognized by the sera of animals and human infected with distinct parasite DTUs, an assumption that was reinforced by our results.

The cross-reactivity of the epitopes among the sera from mice infected with distinct parasite strains is in agreement with an origin hypothesis of the different *T. cruzi* DTUs. In two-way comparisons, the CL Brener and Y strains, the two more phylogenetically related strains, shared a higher number of epitopes (4) compared to Y and Colombiana (1), whereas CL Brener and Colombiana did not share any epitope (Figure 1E).

Interestingly, despite the fact that all of the peptides are derived from the CL Brener genome, a smaller number of epitopes were identified in this strain compared with Y and Colombiana (Figure 1E). We speculate that the co-expression of alleles that encode the polymorphic epitopes in CL Brener may affect the titer of the antibody and/or its affinity for the variant epitopes. For example, the pattern of expression and the reactivity of the polymorphic peptides A6_30_col and B2_30_y (Figures 4B, 4C, 5B, and 5C) suggest that the co-expression of polymorphic epitopes in CL Brener could induce low-affinity antibodies. A similar phenomenon has been described for the polymorphic *T. cruzi* trans-sialidase (TS) multigene family, whereby TS displays a network of B-cell cross-reactive and polymorphic epitopes that delays the generation of high-affinity neutralizing antibodies and hamper an effective elicitation of a humoral response against these proteins [60]. Whether this is a more general adaptive immune evasion strategy that affects antibody affinity maturation, particularly in the case of hybrid strains, remains to be investigated.

Altogether, the results demonstrated that peptide C6_30_cons is a new *T. cruzi* antigen conserved in the majority of DTUs of this parasite. Using this peptide, in association with other *T. cruzi* antigens, may improve the serodiagnosis of Chagas disease. The three polymorphic epitopes identified were able to discriminate among infections caused by the three different *T. cruzi* strains included in this study and, thus, have the potential to be used for the serotyping of infections caused by this parasite. This is the first study on the genomic scale to identify DTU-specific antigens. The genome sequencing of other *T. cruzi* strains will help identify new strain-specific and conserved epitopes and increase the number of antigen candidates for Chagas disease serodiagnosis and serotyping. The development of a robust panel of strain-specific epitopes may allow large-scale epidemiological studies aimed at correlating the infective strain with the variability in clinical outcomes observed in chagasic patients.

## Supporting Information

Figure S1 **Genotyping of** ***T. cruzi*** **isolates infecting chagasic patients from Bolivia.** DNA extracted from blood of chagasic patients was amplified using primers specific to the *T. cruzi* mitochondrial COII gene followed by digestion with the *Alu*I restriction enzyme, as previously described (33), and separation by electrophoresis in 8% polyacrylamide gel. DNA bands were visualized by silver staining. MW, molecular weight; C-, negative control; 1, Genomic DNA of Colombiana clone (TcI); 2, Genomic DNA of Y strain (TcII); 3, Genomic DNA of CL Brener clone (TcVI); 4–9, DNA extracted from blood of chagasic patients. DNA fragments of 264-, 212- and 294-pb characterize TcI, TcII and TcIII-TcVI DTUs, respectively (33).
(TIFF)

Figure S2 **ELISA with the sera from mice infected with different CL Brener strain inocula against conserved and polymorphic peptides.** (A) Peptide C6_30_cons. (B) Peptide A6_30_col. (C) Peptide B2_30_y. (D) Peptide B9_30_cl. The dotted line represents the cutoff value. The solid gray line represents the mean values. C-, uninfected mice. CL Brener 50, mice infected with 50 trypomastigotes of the CL Brener. CL Brener 100, mice infected with 100 trypomastigotes of the CL Brener. CL Brener 1000, mice infected with 1000 trypomastigotes of the CL Brener.
(TIFF)

Figure S3 **Cross-reactivity evaluation of conserved and polymorphic peptides against the sera from mice infected with** ***T. rangeli*** **.** (A) Peptide C6_30_cons. (B) Peptide A6_30_col. (C) Peptide B2_30_y. (D) Peptide B9_30_cl. The dotted line represents the cutoff value. The solid gray line represents the mean values. C-, uninfected mice. *T. rangeli*, mice infected with *T. rangeli*.
(TIFF)

Figure S4 **Cross-reactivity of conserved and polymorphic peptides against sera of patients with cutaneous and visceral leishmaniasis.** (A) Peptide C6_30_cons. (B) Peptide A6_30_col. (C) Peptide B2_30_y. (D) Peptide B9_30_cl. The dotted line represents the cutoff value. The solid gray line represents the mean values. C-, uninfected humans. CL, patients with cutaneous leishmaniasis. VL, patients with visceral leishmaniasis.
(TIFF)

Table S1 **List of primers used to amplify the sequences encoding the epitopes.**
(DOCX)

Table S2 **List of allele-specific primers used in the real-time RT-PCR analysis.**
(DOCX)

Table S3 **Reactivity of the sera from mice infected with different** ***T. cruzi*** **strains against the peptides.**
(XLSX)
