# Supplementary figures and images for: Identification of Strain-Specific B-cell Epitopes in Trypanosoma cruzi Using Genome-Scale Epitope Prediction and High-Throughput Immunoscreening with Peptide Arrays
Source: PLoS Negl Trop Dis. 2013 Oct 31;7(10):e2524. doi: 10.1371/journal.pntd.0002524 (PMC3814679; doi:10.1371/journal.pntd.0002524)

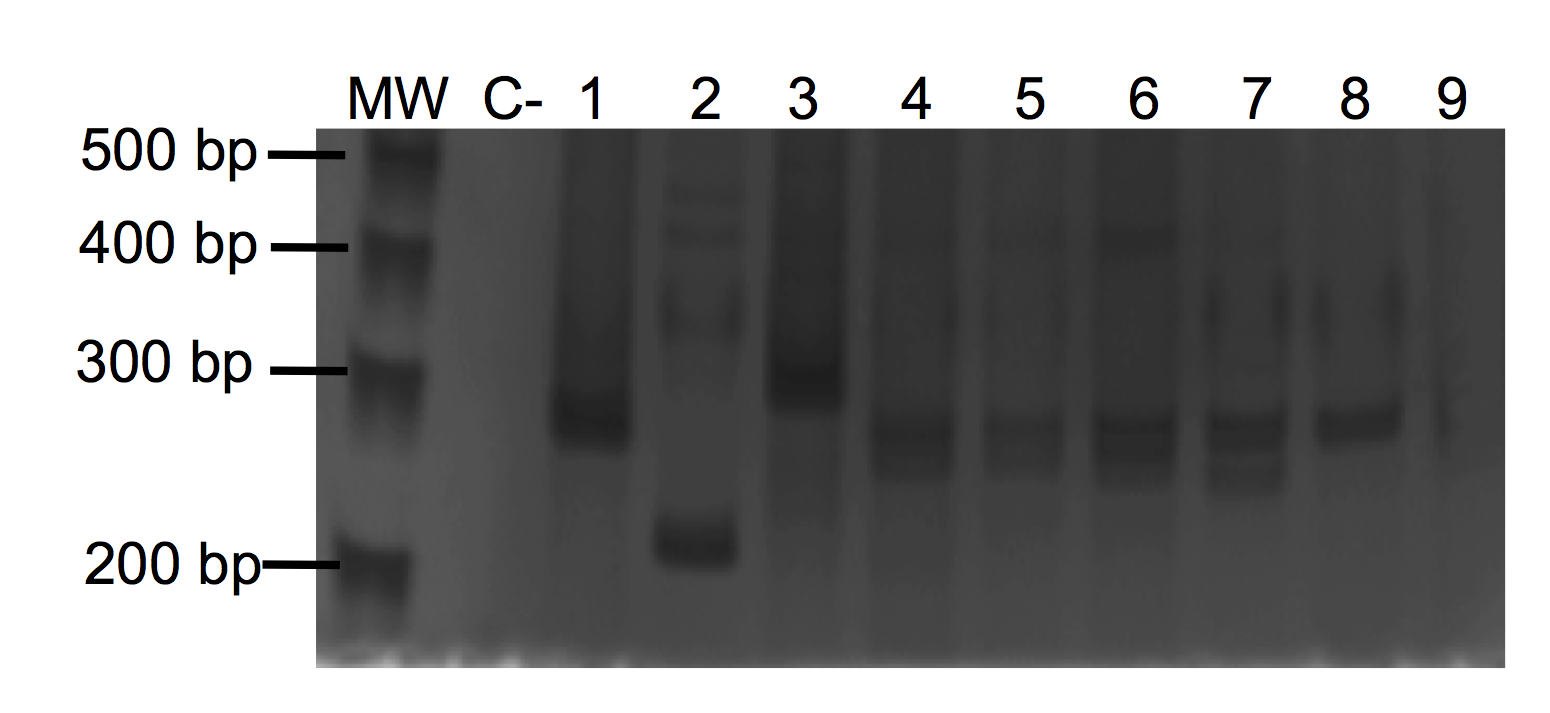

Supplement: Figure S1 — Genotyping of T. cruzi isolates infecting chagasic patients from Bolivia. DNA extracted from blood of chagasic patients was amplified using primers specific to the T. cruzi mitochondrial COII gene followed by digestion with the AluI restriction enzyme, as previously described (33), and separation by electrophoresis in 8% polyacrylamide gel. DNA bands were visualized by silver staining. MW, molecular weight; C-, negative control; 1, Genomic DNA of Colombiana clone (TcI); 2, Genomic DNA of Y strain (TcII); 3, Genomic DNA of CL Brener clone (TcVI); 4–9, DNA extracted from blood of chagasic patients. DNA fragments of 264-, 212- and 294-pb characterize TcI, TcII and TcIII-TcVI DTUs, respectively (33). (TIFF) [file pntd.0002524.s001.tiff]

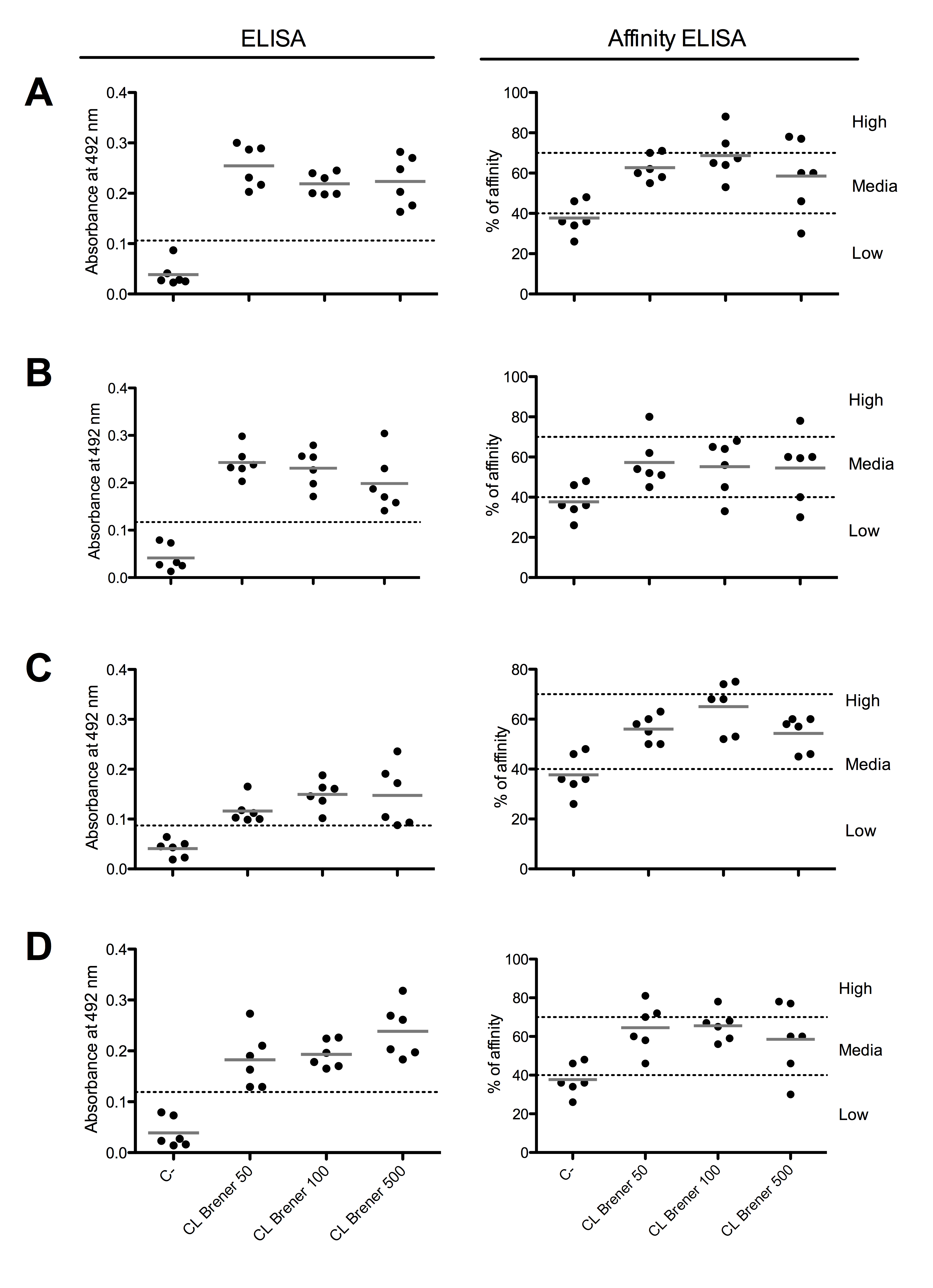

Supplement: Figure S2 — ELISA with the sera from mice infected with different CL Brener strain inocula against conserved and polymorphic peptides. (A) Peptide C6_30_cons. (B) Peptide A6_30_col. (C) Peptide B2_30_y. (D) Peptide B9_30_cl. The dotted line represents the cutoff value. The solid gray line represents the mean values. C-, uninfected mice. CL Brener 50, mice infected with 50 trypomastigotes of the CL Brener. CL Brener 100, mice infected with 100 trypomastigotes of the CL Brener. CL Brener 1000, mice infected with 1000 trypomastigotes of the CL Brener. (TIFF) [file pntd.0002524.s002.tiff]

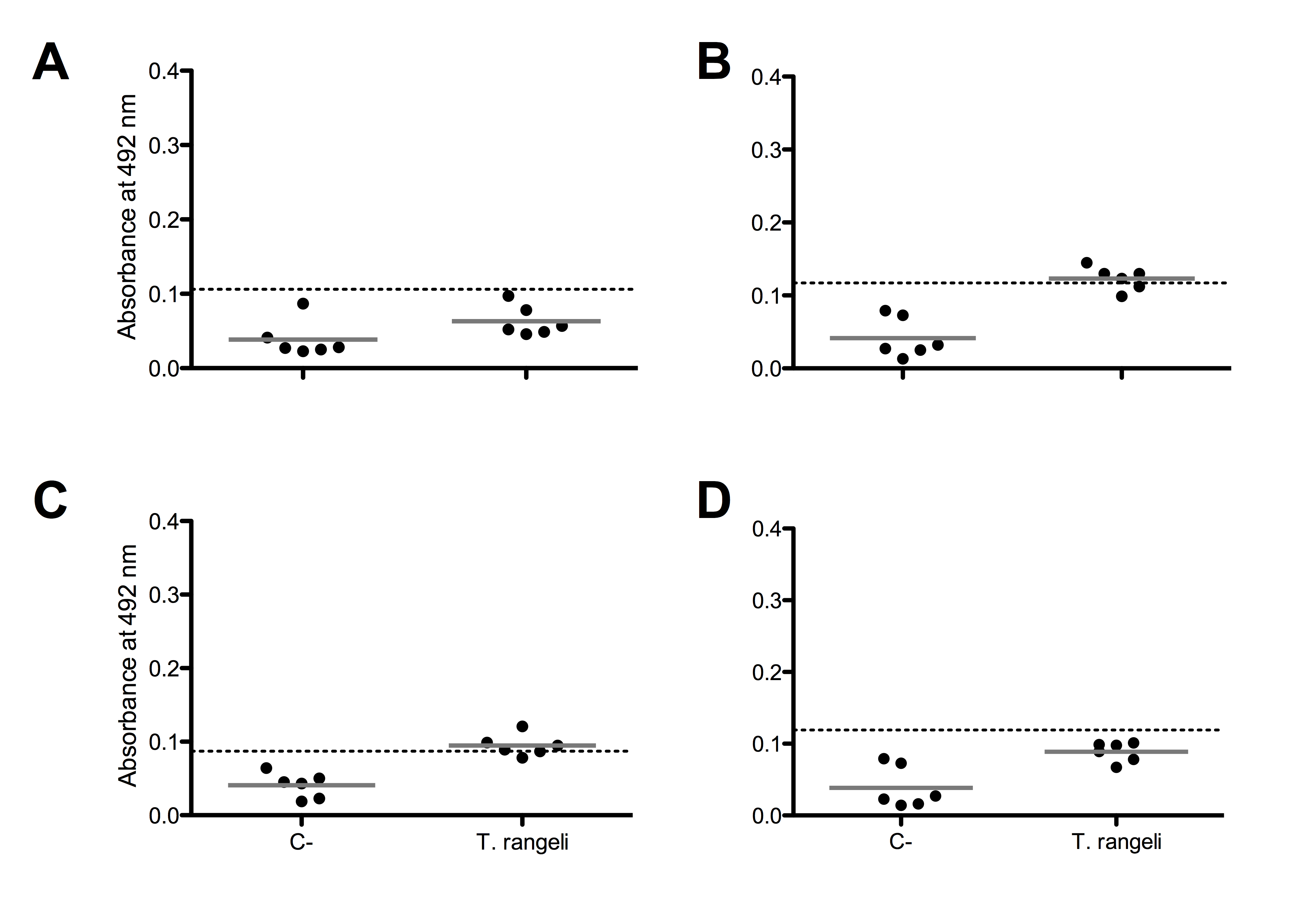

Supplement: Figure S3 — Cross-reactivity evaluation of conserved and polymorphic peptides against the sera from mice infected with T. rangeli . (A) Peptide C6_30_cons. (B) Peptide A6_30_col. (C) Peptide B2_30_y. (D) Peptide B9_30_cl. The dotted line represents the cutoff value. The solid gray line represents the mean values. C-, uninfected mice. T. rangeli, mice infected with T. rangeli. (TIFF) [file pntd.0002524.s003.tiff]

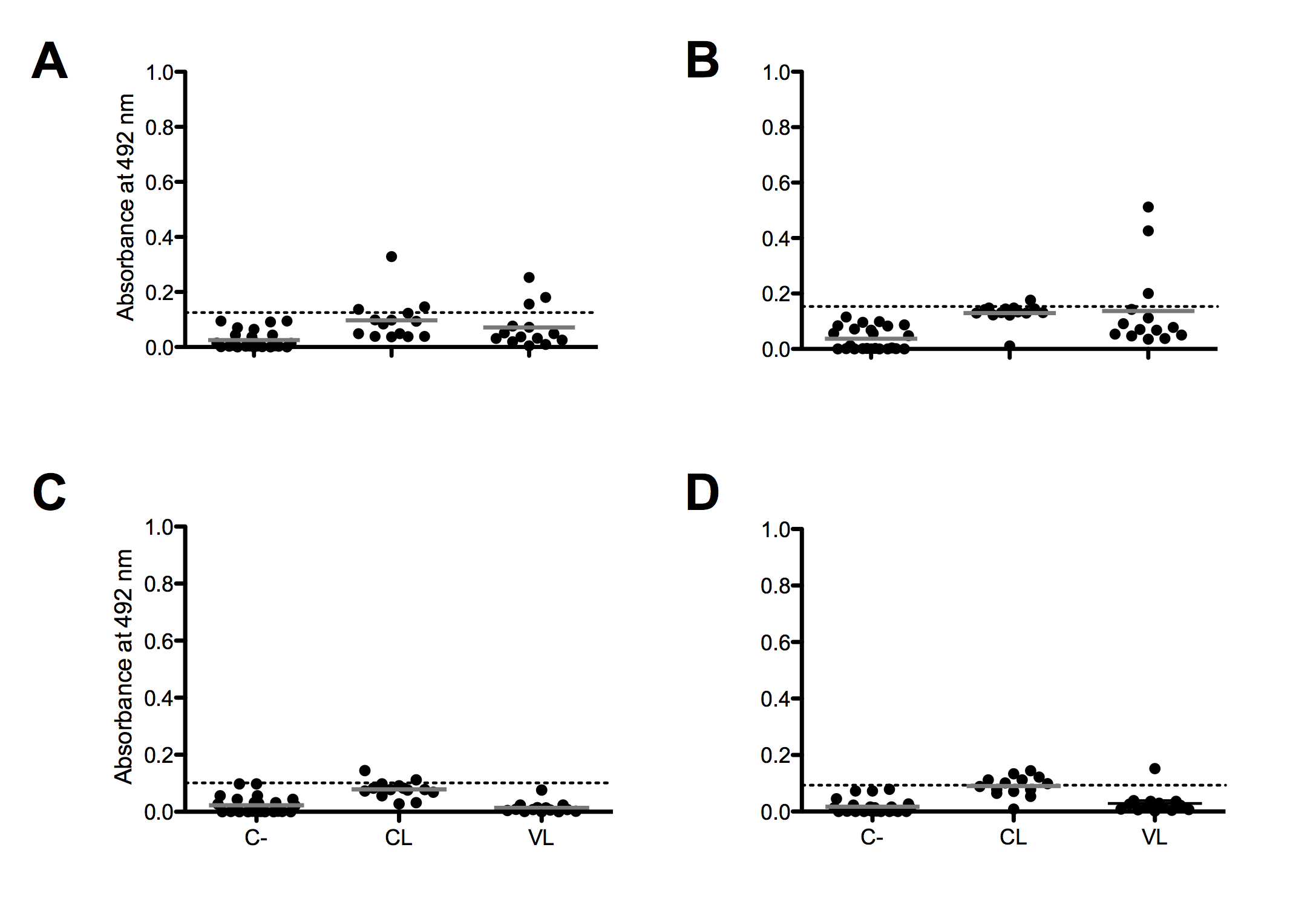

Supplement: Figure S4 — Cross-reactivity of conserved and polymorphic peptides against sera of patients with cutaneous and visceral leishmaniasis. (A) Peptide C6_30_cons. (B) Peptide A6_30_col. (C) Peptide B2_30_y. (D) Peptide B9_30_cl. The dotted line represents the cutoff value. The solid gray line represents the mean values. C-, uninfected humans. CL, patients with cutaneous leishmaniasis. VL, patients with visceral leishmaniasis. (TIFF) [file pntd.0002524.s004.tiff]
