# Supplementary material for: Identification of Strain-Specific B-cell Epitopes in Trypanosoma cruzi Using Genome-Scale Epitope Prediction and High-Throughput Immunoscreening with Peptide Arrays
Source: PLoS Negl Trop Dis. 2013 Oct 31;7(10):e2524. doi: 10.1371/journal.pntd.0002524 (PMC3814679; doi:10.1371/journal.pntd.0002524)
Supplement: Table S1 — List of primers used to amplify the sequences encoding the epitopes. (DOCX) [file pntd.0002524.s005.docx]

**Supplementary Table 1 – List of primers used to amplify the sequences encoding the epitopes.**

| **Epitope** | **Primer Forward** | **Primer Reverse** |
| --- | --- | --- |
| A6 | TGATTTCTCCCTTTGGATGG | AGGAAAAGGTGGGCGAAA |
| B2 | GCACGTCAATATTCCGGTTT | GAGCAGTCGGTGCTTGGTAG |
| B9 | CGTCGCAGTCTACGTCAGTG | TTTGTACGTGTGGGTCGAAA |
| C6 | ATGGAACAGCCGTTGGTATC | TTTCTCTCGCAGTTCCTGCT |
