# Supplementary material for: Identification of Strain-Specific B-cell Epitopes in Trypanosoma cruzi Using Genome-Scale Epitope Prediction and High-Throughput Immunoscreening with Peptide Arrays
Source: PLoS Negl Trop Dis. 2013 Oct 31;7(10):e2524. doi: 10.1371/journal.pntd.0002524 (PMC3814679; doi:10.1371/journal.pntd.0002524)
Supplement: Table S2 — List of allele-specific primers used in the real-time RT-PCR analysis. (DOCX) [file pntd.0002524.s006.docx]

**Supplementary Table 2 – List of allele-specific primers used in the Real Time RT-PCR analysis.**

| **Epitope/Allele** | **Primer Forward** | **Primer Reverse** |
| --- | --- | --- |
| A6/Esmo-like | GCTGACTGTGCCATCCGT | GTGGGGGAGGATTCGCA |
| A6/Non-esmo-like | GCTGACTGTGCCATCCGC | GTGGGGGAGGATTCGCG |
| B2/Esmo-like | GAGTTGTGAGCTTTCTTCGCATT | GCGTCGGGGTCTGTGG |
| B2/Non-esmo-like | GAGTTGTGAGCTCTCTTCGCATC | TGCGTCGGGGTCTGTGA |
| B9/Esmo-like | TGCGGCACGAAAAGCCT | GCCACCTCCACCATGTGC |
| B9/Non-esmo-like | TGCAGCACGAAAAGCCG | AGCCACCTCCACCATGTGT |
| C6/Esmo-like | GGATTCCGGACTTTCTGCG | GAAGGAACGCCACCAAAAAC |
| C6/Non-esmo-like | GCCGTTGGTATCAGCTTTGC | GCTGAAAAATAGCCTGGCGC |
